# Frequency of lumbopelvic malalignment in symptomatic hip instability and impingement – a prospective, diagnostic cohort study

**DOI:** 10.1007/s00402-025-05808-w

**Published:** 2025-04-17

**Authors:** Maximilian Fischer, Lars Nonnenmacher, Andreas Nitsch, Matthias R. Muehler, Andre Hofer, Georgi I. Wassilew

**Affiliations:** 1 Center for Orthopaedics, Trauma Surgery and Rehabilitation Medicine, University Medicine Greifswald, Greifswald, Germany; 2 Department of Radiology and Neuroradiology, University Medicine Greifswald, Greifswald, Germany; 3 Department of Radiology, University of Wisconsin Madison, 600 Highland Ave, Madison, USA

**Keywords:** Developmental dysplasia of the hip, Acetabular retroversion, Lumbopelvic alignment, Spine, Hip-preserving surgery, Lumbopelvic mobility

## Abstract

**Introduction:**

The dynamic lumbopelvic interaction has gained increasing importance in hip-preserving surgery, even though the coexistence of lumbopelvic malalignment with pre-arthritic hip deformities has been poorly studied. This study aimed to examine (I) the frequency of static and functional lumbopelvic malalignment (II) and to compare the lumbopelvic alignment between symptomatic mild to severe hip dysplasia (HD) and impingement-driven acetabular retroversion (AR).

**Methods:**

Sagittal lumbopelvic radiographs were reviewed in standing, relaxed-seated and deep-seated position for pelvic incidence (PI), pelvic tilt (PT), lumbar lordosis (LL), and sacral slope (SS). Static lumbopelvic alignment was classified as “Flatback”, “Normal”, or “Hyperlordotic” and functional lumbopelvic alignment was categorized as “Stiff”, “Normal”, and “Hypermobile”. Static and functional (Δ between the above-mentioned positions) lumbopelvic parameters were compared among HD, borderline hip dysplasia (BHD), and AR.

**Results:**

Ninety-eight patients undergoing hip-preserving surgery for HD (*n* = 47), BHD (*n* = 36), and AR (*n* = 15) were prospectively enrolled. Static lumbopelvic malalignment occurred in 44.9% of patients (44/98), with “Hyperlordotic” alignment being the most frequent (36/44). Additionally, 28.6% of patients (28/98) exhibited functional lumbopelvic malalignment. Static lumbopelvic parameters showed differences between hip instability and impingement, with lower PI (42° vs. 57.3°, *p* = 0.001; 42° vs. 53.7°, *p* = 0.01) and PT (5.6° vs. 15.8°, *p* < 0.001; 5.6° vs. 12.4°, *p* = 0.01) in AR patients compared to HD and BHD in standing position. Moreover, SS was significantly lower in AR (40.9° vs. 50.1°, *p* = 0.02) and BHD (43.8° vs. 50.1°, *p* = 0.05) compared to HD in deep-seated position. Significant differences in functional lumbopelvic parameters were observed only between HD and BHD in PT (Δ standing – deep-seated position, 7.1° vs. -1.2°, *p* = 0.04).

**Conclusion:**

Static and functional lumbopelvic malalignment is prevalent in patients with pre-arthritic hip deformities. While static lumbopelvic parameters vary between instability- and impingement-driven hip deformities, functional lumbopelvic alignment is quite similar among HD, BHD, and AR.

## Introduction

There is rising interest in lumbopelvic relations in hip-preserving surgery, accelerated by recent findings of significant differences in lumbopelvic parameters across various pre-arthritic hip deformities [1–4]. For both instability-driven hip dysplasia (HD) and impingement-causing acetabular retroversion (AR), increased pelvic tilting on static radiographs has been linked to compensating for insufficient femoral head coverage in HD, as well as to initiating an extended cross over sign in AR [5–7].

Focusing on static lumbopelvic radiographs in patients with pre-arthritic hip diseases, changes in pelvic tilt (PT) have also been described between the in-hospital typical supine and standing positions [8–10]. However, with respect to the dynamic interaction between the spine, pelvis, and femur, lumbopelvic metrics differ substantially across various daily activity positions [11, 12]. Consequently, recent studies on patients with adult spinal deformities, a highly prevalent degenerative disease in older patients, have highlighted the importance of 3D or functional radiographic lumbopelvic assessment as relevant factor for acetabular orientation and postoperative outcomes [13, 14]. Furthermore, altered lumbopelvic alignment has been associated with changes in acetabular orientation during gait in such patient cohort [15].

Consequently, lumbopelvic alignment in younger patients undergoing hip-preserving surgery should also be studied functionally across various positions (standing, relaxed-seated, deep-seated) to precisely mimic daily activities. This would allow for a deeper and detailed understanding of static and functional lumbopelvic alignment in this patient cohort [16].

Furthermore, the coexistence of pre-arthritic hip diseases with lumbopelvic malalignment may affect the postoperative outcomes in this patient cohort. Although, the coexistence and functional influence of spinal malalignment on hip osteoarthritis has already been described, this topic has been poorly studied in patients with pre-arthritic hip diseases [17, 18].

Thus, the purpose of this prospective, monocenter diagnostic cohort study was (I) to report the frequency of static and functional lumbopelvic malalignment in a representative cohort of patients with pre-arthritic hip deformities; and (II) to compare the static and functional lumbopelvic alignment in hip instability due to mild to severe HD as well as impingement-driven AR.

## Patients and methods

### Study design

A prospective, institutional review board-approved, diagnostic cohort study was performed in one tertiary institution (Table 1). Patients presented with symptomatic and refractory hip pain lasting more than 6 months and operative hip-preserving therapy was initiated between 01/2024 and 06/2024. All patients gave written informed consent prior to inclusion. Ethics approval (BB099/20a) was obtained from the local independent ethics committee (IEC) of the University Medicine Greifswald according to the World Medical Association Declaration of Helsinki.

**Table 1:** Patient characteristics and radiographic acetabular morphology. HD hip dysplasia, BHD borderline hip dysplasia, AR acetabular retroversion, LCEA lateral center-edge angle, AI acetabular inclination, AWI anterior wall index, PWI posterior wall index

	Total (*N* = 98)		HD (*N* = 47)	BHD *N* = 36)	AR (*N* = 15)
Mean Age, yrs. (SD)	30.3 (7.7)		31.8 (7.3)	31.5 (7.7)	23.6 (4.7)
Female (%)	77.5		85.1	72.2	60
Mean LCEA, ° (SD)	*17.8* *(9.1)*		*10.6* *(6.4)*	*20.8* *(1.8)*	*30.7* *(3.4)*
Mean AI, ° (SD)	*12.1* *(7.5)*		*17.8* *(4.8)*	*9.8* *(2.7)*	*0.64* *(2.6)*
Mean AWI (SD)	*0.38* *(0.16)*		*0.31* *(0.1)*	*0.41* *(0.12)*	*0.62* *(0.17)*
Mean PWI (SD)	*0.83* *(0.15)*		*0.84* *(0.17)*	*0.85* *(0.14)*	*0.75* *(0.1)*

### Radiographic assessment

Each patient underwent sagittal lumbopelvic radiographs including the lumbar spine, pelvis and proximal femur. This included a consecutive series radiographs in standing, relaxed-seated (hip flexion 90°, femurs parallel to the floor) and deep-seated position (maximal forward leaning, femurs parallel to the floor).

Radiographs were reviewed for pelvic incidence (PI), lumbar lordosis (LL), sacral slope (SS) and pelvic tilt (PT) in all above-mentioned positions.

**Fig. 1:**
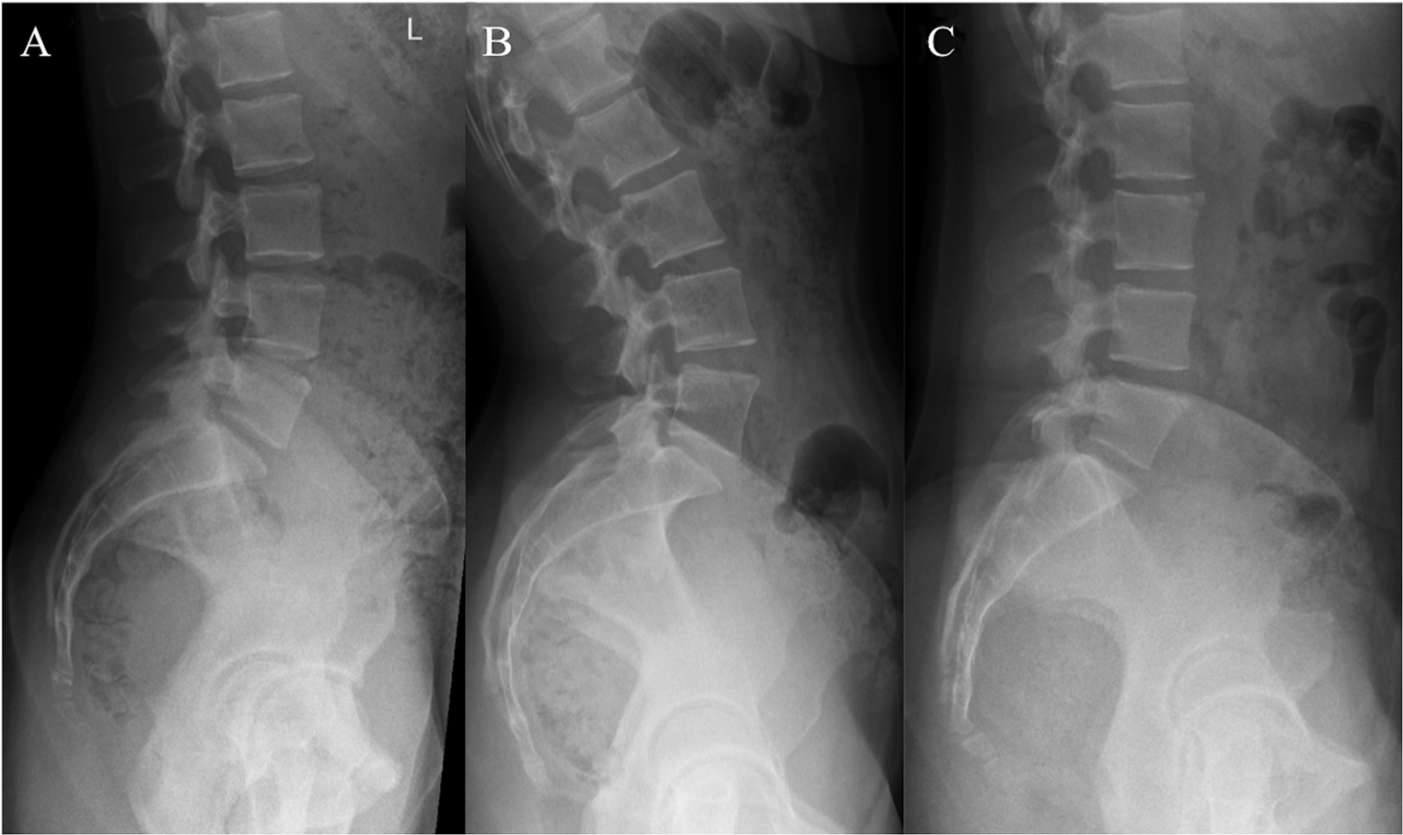
Representative radiographs with different types of static lumbopelvic alignment (A Normal – PI-LL -10° − 10°, B Hyperlordotic – PI-LL < -10°, C Flatback – PI-LL > 10°). PI pelvic incidence, LL lumbar lordosis

Static lumbopelvic alignment was classified on standing lumbopelvic radiographs as “Flatback”, “Normal”, or “Hyperlordotic” using the PI-LL mismatch with a cutoff of ± 10° Fig. 1) [19].

**Fig. 2:**
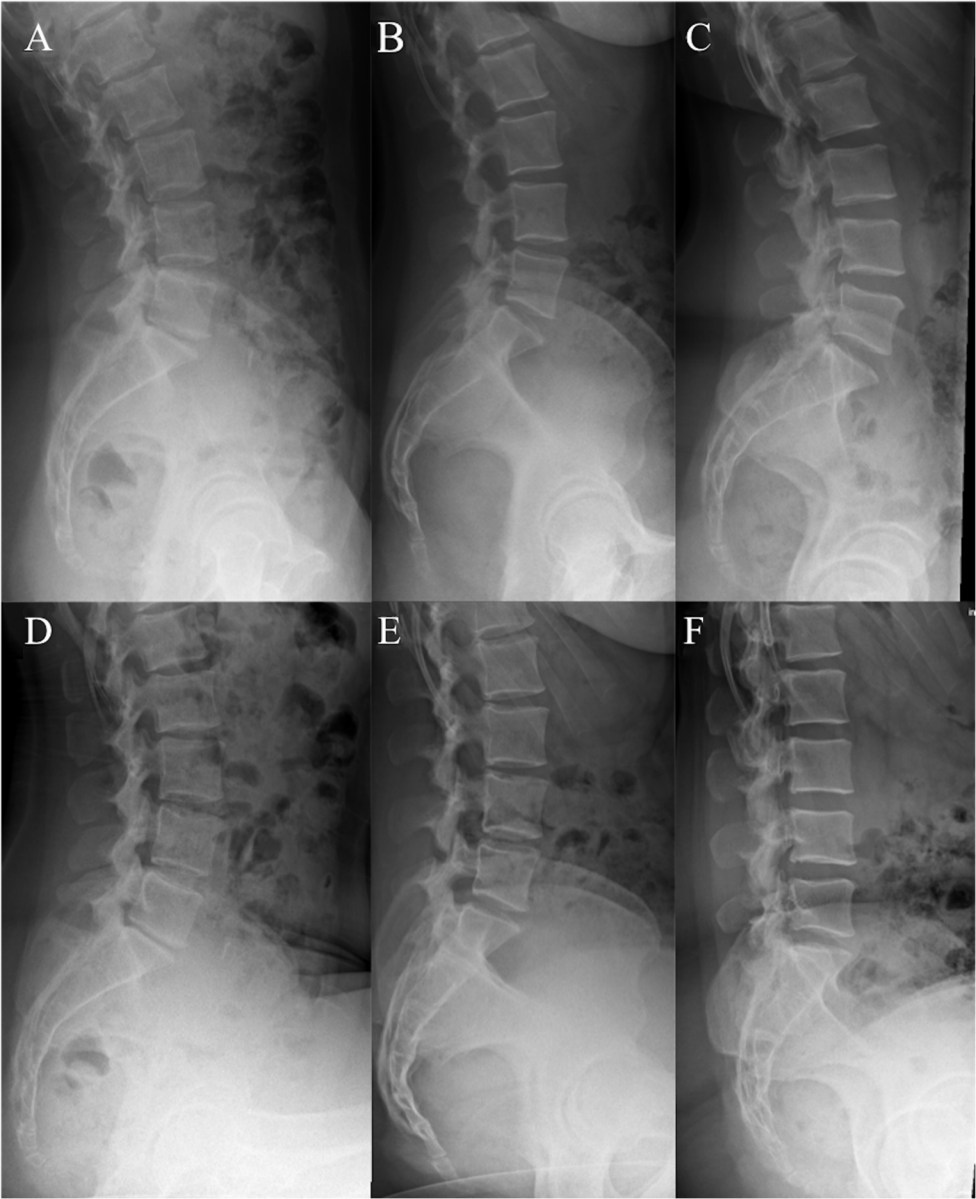
Representative radiographs with different types of functional lumbopelvic alignment in standing and relaxed-seated position (A/D Stiff - Δ PT < 10°, B/E Normal - Δ PT 10° − 30°, C/F Hypermobile - Δ PT > 30°). PT pelvic tilt

Patients functional lumbopelvic alignment were classified in accordance to their change (Δ value) in PT between standing and relaxed-seated position as “Stiff”, “Normal”, and “Hypermobile” (Fig. 2) [20].

**Fig. 3:**
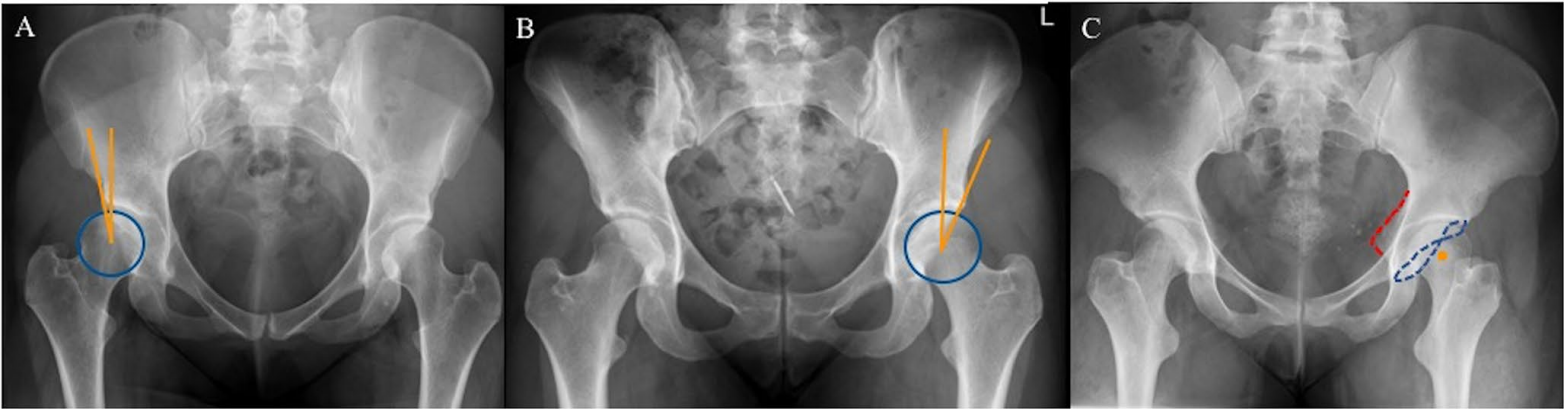
Representative anteroposterior pelvic radiographs of (**A**) hip dysplasia – LCEA < 18° - orange line (**B**) borderline hip dysplasia LCEA 18° − 25° - orange line, and (**C**) acetabular retroversion - red sciatic spine sign, blue crossing-over sign, orange posterior wall sign. LCEA lateral center-edge angle

Acetabular morphology was assessed on anteroposterior pelvic radiographs as well as axial and faux-profile femoral views for Tönnis osteoarthritis grade (< 2), lateral center-edge angle (LCEA), acetabular inclination (AI), anterior- and posterior wall index (AWI/PWI) and signs of acetabular retroversion (Table 1). HD was classified using a LCEA cut-off value of < 18° (Fig. 3A), while BHD was defined through a LCEA between 18° − 25° (Fig. 3B). Acetabular retroversion was defined with all three signs of acetabular retroversion available on anteroposterior pelvic radiographs (crossing-over, posterior wall sign, sciatic spine sign) combined with a LCEA > 25° (Fig. 3C) [21].

### Data collection and statistical analysis

Descriptive statistics were used to summarize the patient characteristics and lumbopelvic measurements. Statistical analysis were performed using SPSS (version 29, IBM, Armonk, NY, USA). Normal distribution was tested using the *Shapiro-Wilk test*. Differences in static and functional lumbopelvic alignment between HD, BHD, and AR were tested for statistical significance by analysis of variance (*ANOVA*). A p-value less than 0.05 was considered statistically significant.

## Results

### Frequency of static and functional lumbopelvic malalignment

Ninety eight patients (77.5% female) with symptomatic HD (*n* = 47), BHD (*n* = 36) and AR (*n* = 15) were included in this study. The mean age was 30.3 ± 7.7 years (Table 1).

**Fig. 4:**
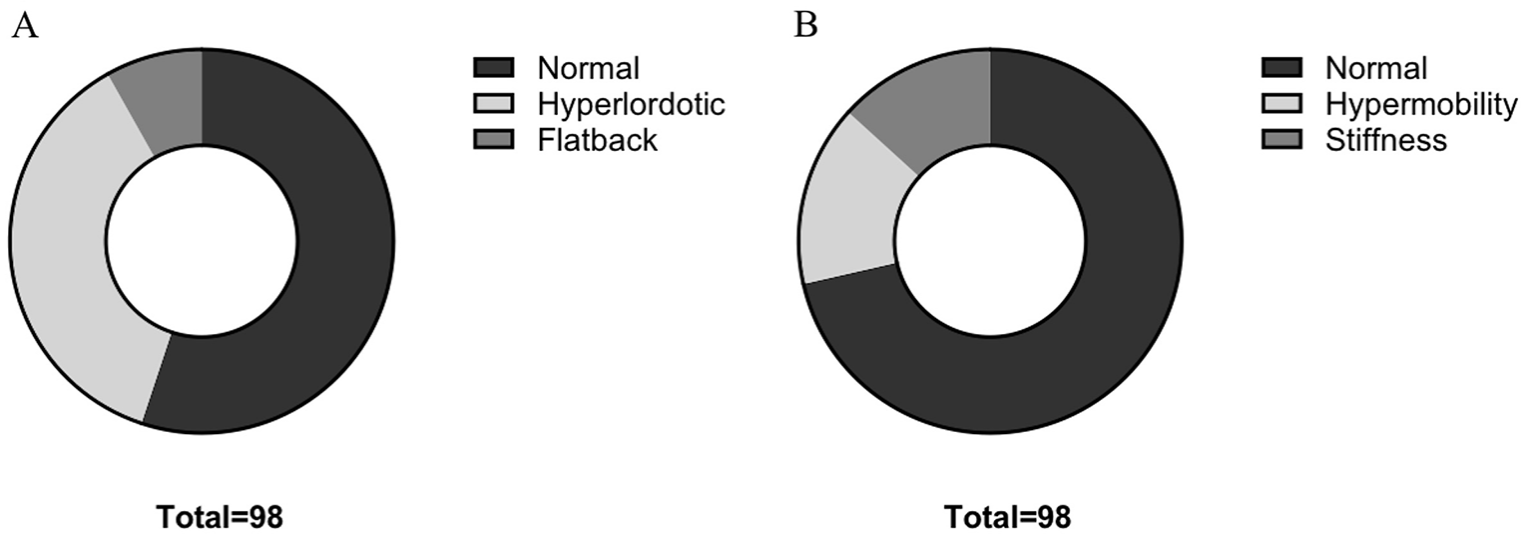
Frequency of static (**A**) and functional (**B**) lumbopelvic malalignment

Static lumbopelvic malalignment, defined as “Flatback” or “Hyperlordotic” in standing position, occurred in 44.9% of patients (44/9) Fig. 4A). Overall, “Hyperlordosis” was the most frequent (36/44).

“Hypermobility” occurred in 15.3% of patients (15/98) and 13.3% of patients exhibited functional lumbopelvic “Stiffness” (13/9) Fig. 4B).

Static lumbopelvic parameters showed significant differences in standing and deep-seated position between joint instability and impingement (Table 2).

In standing position, the mean PI (42° vs. 57.3°, *p* = 0.001; 42° vs. 53.7°, *p* = 0.01) and the mean PT (5.6° vs. 15.8°, *p* < 0.001; 5.6° vs. 12.4°, *p* = 0.01) was significantly lower in AR compared to HD and BHD. In deep-seated position, the mean SS showed significantly lower values in BHD (43.8° vs. 50.1°, *p* = 0.05) and AR (40.9° vs. 50.1°, *p* = 0.02) compared to HD.

**Table 2:** Static lumbopelvic parameters in standing, relaxed-seated and deep seated position. HD hip dysplasia, BHD borderline hip dysplasia, AR acetabular retroversion, # significance difference compared to HD, § significance difference compared to BHD, *p* < 0.05

	HD (*N* = 47)	BHD (*N* = 36)	AR (*N* = 15)
Mean Pelvic incidence, ° (SD)	*57.3* *(13.4)*	*53.7* *(11.5)*	***42.0 # §*** ***(7.8)***
Mean Lumbar lordosis - Standing, ° (SD)	*59.7* *(10.8)*	*60.2* *(10.9)*	*53.9* *(12.1)*
Mean Sacral Slope - Standing, ° (SD)	*42.0* *(10.3)*	*40.2* *(7.9)*	*36.5* *(6.9)*
Mean Pelvic tilt - Standing, ° (SD)	*15.8* *(7.9)*	*12.4* *(7.2)*	***5.6 # §*** ***(6.8)***
Mean Lumbar lordosis – Relaxed- Seated, ° (SD)	*33.5* *(11.7)*	*30.4* *(11.1)*	*25.3* *(15.3)*
Mean Sacral Slope – Relaxed- Seated ° (SD)	*25.6* *(8.8)*	*21.4* *(8.1)*	*16.7* *(8.5)*
Mean Pelvic tilt – Relaxed- Seated ° (SD)	*33.2* *(12)*	*33.4* *(11.6)*	*28.1* *(11)*
Mean Lumbar lordosis – Deep Seated, ° (SD)	*0.3* *(10.5)*	*-2.1* *(10.5)*	*-4.3* *(12.9)*
Mean Sacral Slope – Deep Seated, ° (SD)	*50.1* *(11.6)*	***43.8 #*** ***(11)***	***40.9 #*** ***(10.3)***

### Functional lumbopelvic alignment

**Fig. 5:**
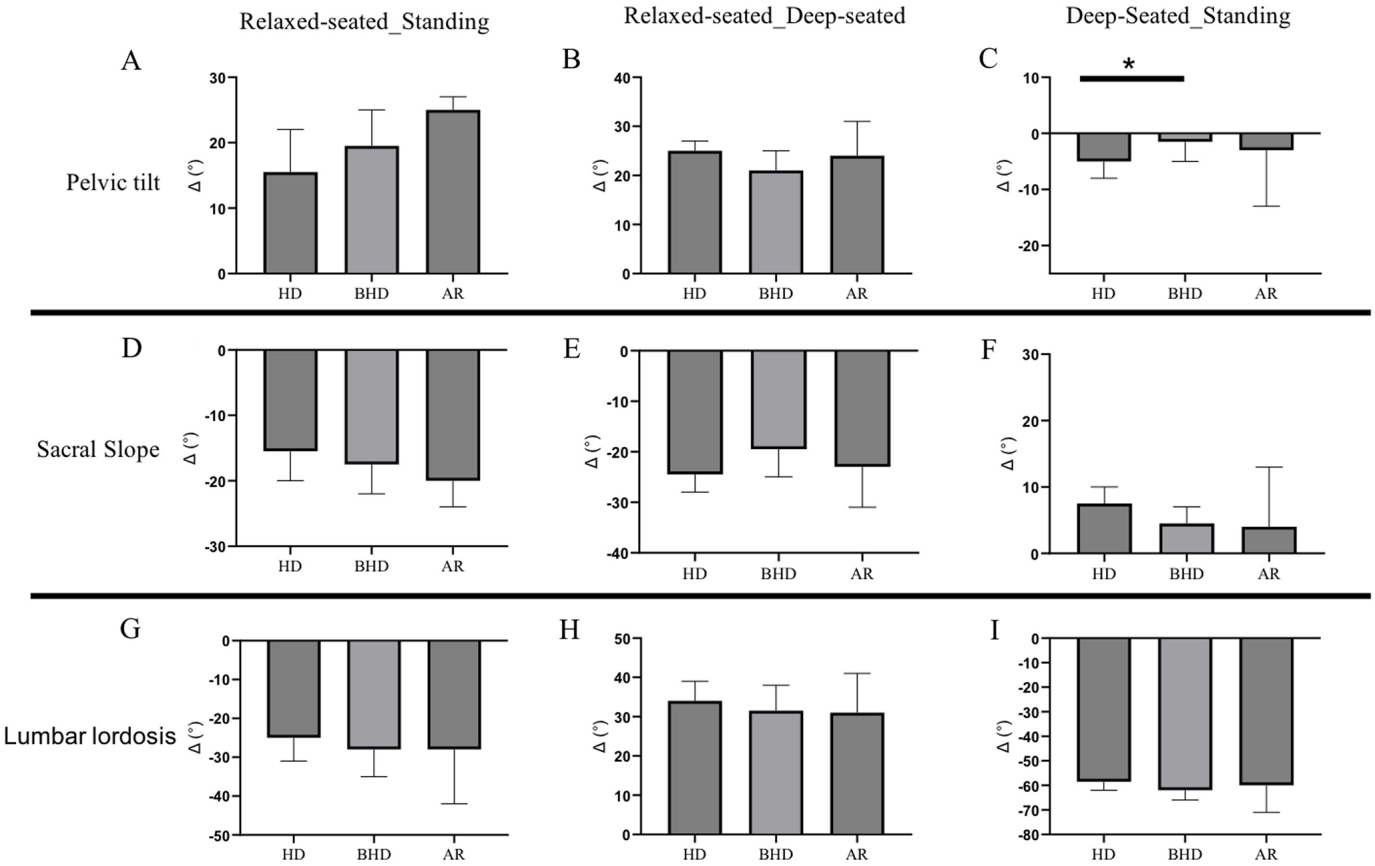
Functional lumbopelvic alignment between standing, relaxed-seated and deep-seated position. HD hip dysplasia, BHD borderline hip dysplasia, AR acetabular retroversion, Median with 95% confidence interval, * *p* < 0.05

Functional lumbopelvic alignment was assessed by the change in PT-, SS- and LL-angle between the standing, relaxed-seated and deep-seated position (Fig. 5). The mean change in PT angle between the standing and the deep-seated position was significantly higher in HD compared to BHD (-7.1° vs. -1.2°, *p* = 0.04) (Fig. 5C). While a wide interindividual variability occurred, no additional functional lumbopelvic parameter reached statistical significance across the analyzed acetabular morphologies.

## Discussion

The main findings of this study were; (I) static and functional lumbopelvic malalignment is prevalent in patients undergoing hip-preserving surgery and (II) static lumbopelvic alignment significantly vary between instability- and impingement-driven hip deformities, while functional lumbopelvic alignment is quite similar between HD, BHD and AR.

Hip preserving interventions are performed in a wide range of joint morphologies with different pathomorphological features and have gained considerable importance in recent decades [22–24]. Nevertheless, the lumbopelvic interaction has largely been overlooked and previous investigations on this topic in pre-arthritic hip deformities were mainly focused on isolated acetabular morphologies or were conducted in asymptomatic patient cohorts on static radiographs [25–27]. In contrast, the current study included symptomatic patients with instability- and impingement-driven pre-arthritic hip deformities, as well as a comprehensive static and functional lumbopelvic radiographic assessment. Considering these, this study contributes to an improved understanding of lumbopelvic interaction in hip-preserving patient cohorts.

In the current study cohort, nearly half of the patients exhibited static lumbopelvic malalignment, with “hyperlordotic” alignment in the majority of cases. It has been shown that lumbar lordosis is influenced by age with greater lumbar lordosis in young patients [28]. The current study were conducted in a typically young cohort with a mean age of 30 years, which is in line with other cohorts undergoing hip-preserving interventions [29–31]. Comparing these results with the literature on hip osteoarthritis patients, Buckland et al. reported a prevalence of 41% for static lumbopelvic malalignment in their cohort, which aligns with the current findings on young patients with pre-arthritic hip deformities [18]. Consequently, the lumbopelvic alignment should be considered even in patients undergoing hip-preserving surgery.

Besides the high prevalence of lumbopelvic malalignment in the current patient cohort, the study found significant differences in static lumbopelvic parameters between HD, BHD, and AR. Comparing these results with the existing literature, the current study confirms and extends the knowledge on lumbopelvic alignment by including a functional lumbopelvic assessment. Previous studies has mainly shown differences in static spinopelvic parameters but did not report on functional changes in different postures of daily living [2, 3]. The importance of functional lumbopelvic assessment has been emphasized in patients with adult spinal deformities for surgical planning, as it is associated with acetabular metrics and postoperative outcomes. However, these studies included older patients with primarily spinal symptoms, in contrast to younger symptomatic patients with hip deformities [13, 15]. Consequently, an adapted functional lumbopelvic assessment has been applied to the cohort of young patients undergoing hip-preservation surgery in the current study.

With respect to static lumbopelvic parameters, the current study found significant lower PI and PT in symptomatic patients with hip impingement compared to those with joint instability, which is in line with results from previous studies [2, 26]. Aiming to improve the treatment strategy in those patients, the current study compared a symptomatic cohort undergoing hip-preserving surgery. In this context it is worth noting, that the radiographic acetabular appearance could be substantially affected by changes in PT. For instance, an increased PT can cause an extended crossing-over sign on pelvic radiographs, which may affects the surgical recommendation [9, 32].

Besides differences in static lumbopelvic parameters, functional lumbopelvic alignment was quite similar between HD, BHD and AR, even when the current study found an isolated significant difference in functional PT between HD and BHD. In contrast, Heimann et al. reported no differences in PT across various pre-arthritic hip deformities [3]. In addition to methodological differences in measurement of PT, the functional assessment may contribute to this conflicting results, remaining at risk for individual performance bias, particularly in positions of peak motion.

Connecting the current study results on static and functional lumbopelvic alignment to studies focusing on patient symptoms and clinical outcomes emphasize the importance of considering lumbopelvic alignment in hip-preserving surgery. For instance, recent data illustrated that coexisting lumbar diseases are associated with an increased risk of hip arthroscopy failure whereas a lower PI was connected to improved postoperative results [33–36]. Furthermore, patients with symptomatic anterior hip impingement were found to exhibit less lumbar mobility compared to asymptomatic controls. Thus, a higher pelvic mobility may represent a compensatory mechanism to achieve an impingement-free range of motion [37, 38]. Therefore, the functional lumbopelvic assessment illustrated in the current study may serve as a useful preoperative tool for planning hip-preserving surgery.

### Limitations

Nevertheless, several limitations in this study must be discussed. First, even when the current study reported on a representative study cohort with various pre-arthritic hip deformities, the findings may not be generalizable to other populations due to the study being conducted at a single university center with a selected patient cohort. Furthermore, this study only included lumbopelvic and acetabular metrics, without assessing femoral deformities that may contribute to lumbopelvic alignment. In this way, it has to be mentioned, that the acetabular metrics were reviewed on 2D pelvic radiographs without 3D calculation of acetabular ante-/ retroversion on pelvic CT or MRI scans. Next, while assessing the functional lumbopelvic alignment in different positions of daily living, a detailed description of lumbopelvic kinematics is missing and needs to be studied in further analyses. In this respect, functional radiographic assessments remain at risk for an individual performance bias. Lastly, this study did not report patient-reported outcomes, which would further improve the understanding of lumbopelvic alignment in these patient cohorts.

## Conclusion

Overall, the present study revealed, that static and functional lumbopelvic malalignment is prevalent in patients undergoing hip-preserving surgery. Static lumbopelvic parameters vary between instability- and impingement-driven hip deformities across various positions of daily living. Further studies including patient-reported outcomes are needed to close the gap between postoperative results and lumbopelvic alignment in hip-preserving surgery.

## Data Availability

No datasets were generated or analysed during the current study.

## References

[1] Eftekhary N et al (2019) A systematic approach to the hip-spine relationship and its applications to total hip arthroplasty. Bone Joint J 101–B(7):808–816 10.1302/0301-620X.101B7.BJJ-2018-1188.R1 31256658

[2] Lerch TD et al (2021) Lower pelvic tilt, lower pelvic incidence, and increased external rotation of the Iliac wing in patients with femoroacetabular impingement due to acetabular retroversion compared to hip dysplasia. Bone Joint Open 2(10):813–824 34619033 10.1302/2633-1462.210.BJO-2021-0069.R1 PMC8558448

[3] Heimann AF et al (2024) *Influence of acetabular and femoral morphology on pelvic tilt.* The Bone & Joint Journal, 106-B(5 Supple B): pp. 3–10 10.1302/0301-620X.106B5.BJJ-2023-0690.R1 38688494

[4] Vaswani R et al (2022) Hip–Spine syndrome in the nonarthritic patient. Arthroscopy: J Arthroscopic Relat Surg 38(10):2930–2938 10.1016/j.arthro.2022.04.015 35550420

[5] Siebenrock K, Kalbermatten D, Ganz R (2003) Effect of pelvic Tilt on acetabular retroversion: a study of Pelves from cadavers. Clin Orthop Relat Res (1976–2007) 407:241–248 10.1097/00003086-200302000-00033 12567152

[6] Fukushima K et al (2018) Relationship between spinal sagittal alignment and acetabular coverage: a patient-matched control study. Arch Orthop Trauma Surg 138:1495–1499 29971509 10.1007/s00402-018-2992-z

[7] Ren P et al (2020) Sagittal spinal-pelvic alignment in patients with Crowe type IV developmental dysplasia of the hip. BMC Musculoskelet Disord 21:1–6 10.1186/s12891-020-03717-0 PMC7568827 33069234

[8] Tachibana T et al (2019) Does acetabular coverage vary between the supine and standing positions in patients with hip dysplasia?? Clin Orthop Relat Res 477(11):2455–2466 31389893 10.1097/CORR.0000000000000898 PMC6903855

[9] Jenkinson MRJ et al (2022) Pelvic Tilt from supine to standing in patients with symptomatic acetabular retroversion of the hip. Bone Joint J 104–B(7):786–791 10.1302/0301-620X.104B7.BJJ-2021-1721.R1 35775175

[10] Jenkinson MRJ et al (2024) Acetabular retroversion: functional or anatomical? Bone Joint J 106–B(2):128–135 38295849 10.1302/0301-620X.106B2.BJJ-2023-0706.R1

[11] Langston J et al (2018) Risk factors for increased sagittal pelvic motion causing unfavourable orientation of the acetabular component in patients undergoing total hip arthroplasty. Bone Joint J 100(7):845–852 29954196 10.1302/0301-620X.100B7.BJJ-2017-1599.R1

[12] Heckmann ND et al (2024) *Excessive posterior pelvic tilt from preoperative supine to postoperative standing after total hip arthroplasty.* The Bone & Joint Journal, 106-B(3 Supple A): pp. 74–80 10.1302/0301-620X.106B3.BJJ-2023-0835.R2 38423083

[13] Mekhael E et al (2023) Functional assessment using 3D movement analysis can better predict health-related quality of life outcomes in patients with adult spinal deformity: a machine learning approach. Front Surg 10 10.3389/fsurg.2023.1166734 PMC10189154 37206356

[14] Mekhael M et al (2021) Toward Understanding the underlying mechanisms of pelvic Tilt reserve in adult spinal deformity: the role of the 3D hip orientation. Eur Spine J 30:2495–2503 10.1007/s00586-021-06778-4 33638719

[15] Assi A et al (2023) ASD with high pelvic retroversion develop changes in their acetabular orientation during walking. Brain Spine 3:101752 37383434 10.1016/j.bas.2023.101752 PMC10293306

[16] Lazennec JY, Brusson A, Rousseau MA (2013) Lumbar-pelvic-femoral balance on sitting and standing lateral radiographs. Orthop Traumatol Surg Res 99(1 Suppl):S87–103 23375267 10.1016/j.otsr.2012.12.003

[17] Buckland AJ et al (2015) Acetabular anteversion changes due to spinal deformity correction: bridging the gap between hip and spine surgeons. JBJS 97(23):1913–1920 10.2106/JBJS.O.00276 26631991

[18] Buckland AJ et al (2020) Prevalence of sagittal spinal deformity among patients undergoing total hip arthroplasty. J Arthroplasty 35(1):160–165 31493962 10.1016/j.arth.2019.08.020

[19] Schwab FJ et al (2013) Radiographical spinopelvic parameters and disability in the setting of adult spinal deformity: a prospective multicenter analysis. Spine 38(13):E803–E812 23722572 10.1097/BRS.0b013e318292b7b9

[20] Innmann MM et al (2020) How can patients with mobile hips and stiff lumbar spines be identified prior to total hip arthroplasty? A prospective, diagnostic cohort study. J Arthroplast 35(6):S255–S261 10.1016/j.arth.2020.02.029 32205003

[21] Lerch TD et al (2022) Diagnosis of acetabular retroversion: three signs positive and increased retroversion index have higher specificity and higher diagnostic accuracy compared to isolated positive cross over sign. Eur J Radiol Open 9:100407 35242888 10.1016/j.ejro.2022.100407 PMC8885617

[22] Hanke MS et al (2020) Hip preservation. EFORT Open Reviews 5(10):630–640 33204506 10.1302/2058-5241.5.190074 PMC7608518

[23] Sienko A, Ekhtiari S, Khanduja V (2023) The growth of hip preservation as a speciality. Knee Surg Sports Traumatol Arthrosc 31(7):2540–2543 37045973 10.1007/s00167-023-07409-9

[24] Zusmanovich M et al (2022) The incidence of hip arthroscopy in patients with femoroacetabular impingement syndrome and labral pathology increased by 85% between 2011 and 2018 in the united States. Arthroscopy: J Arthroscopic Relat Surg 38(1):82–87 10.1016/j.arthro.2021.04.049 33964383

[25] Okuzu Y et al (2019) Hip-spine syndrome: acetabular anteversion angle is associated with anterior pelvic Tilt and lumbar hyperlordosis in patients with acetabular dysplasia: a retrospective study. JBJS Open Access 4(1):e0025 31161147 10.2106/JBJS.OA.18.00025 PMC6510466

[26] Hellman MD et al (2017) Femoroacetabular impingement and pelvic incidence: radiographic comparison to an asymptomatic control. Arthroscopy: J Arthroscopic Relat Surg 33(3):545–550 10.1016/j.arthro.2016.08.033 27939070

[27] Weinberg DS et al (2016) Radiographic signs of femoroacetabular impingement are associated with decreased pelvic incidence. Arthroscopy: J Arthroscopic Relat Surg 32(5):806–813 10.1016/j.arthro.2015.11.047 26947741

[28] Dreischarf M et al (2014) Age-related loss of lumbar spinal lordosis and mobility–a study of 323 asymptomatic volunteers. PLoS ONE 9(12):e116186 25549085 10.1371/journal.pone.0116186 PMC4280226

[29] Andronic O et al (2023) Factors influencing patient-reported outcomes following periacetabular osteotomy and open osteochondroplasty in the setting of borderline hip dysplasia: a retrospective study with minimum follow-up of five years. Bone Joint J 105(7):735–742 37391200 10.1302/0301-620X.105B7.BJJ-2022-1058.R2

[30] Minkara AA et al (2019) Systematic review and meta-analysis of outcomes after hip arthroscopy in femoroacetabular impingement. Am J Sports Med 47(2):488–500 29373805 10.1177/0363546517749475

[31] Larsen JB et al (2020) 14-year hip survivorship after periacetabular osteotomy: a follow-up study on 1,385 hips. Acta Orthop 91(3):299–305 32106751 10.1080/17453674.2020.1731159 PMC8023930

[32] Ross J et al (2013) The influence of pelvic Tilt on common acetabular parameters and range of motion in patients with femoroacetabular impingement (FAI). Arthroscopy: J Arthroscopic Relat Surg 29(12, Supplement):e203

[33] Chatterjee A et al (2024) Patients with a history of lumbar fusion have a greater risk of revision arthroscopy and conversion to total hip arthroplasty after primary hip arthroscopy. The Journal of Arthroscopic & Related Surgery, Arthroscopy 10.1016/j.arthro.2024.08.026 39216680

[34] Torabian KA et al (2024) The effect of pelvic incidence on outcomes after hip arthroscopy for femoroacetabular impingement and acetabular labral tears. Am J Sports Med 52(3):631–642 38369972 10.1177/03635465231219261 PMC10905981

[35] Feingold JD et al (2023) The outcome of hip arthroscopy in the setting of lumbar spine disease is beneficial, yet limited: A systematic review of existing evidence. Arthroscopy: J Arthroscopic Relat Surg 39(6):1568–1583 10.1016/j.arthro.2022.09.014 36191731

[36] Krishnamoorthy VP et al (2019) Radiographic prevalence of sacroiliac joint abnormalities and clinical outcomes in patients with femoroacetabular impingement syndrome. Arthroscopy 35(9):2598–2605e1 31500745 10.1016/j.arthro.2019.03.030

[37] Fader RR et al (2018) The role of lumbar lordosis and pelvic sagittal balance in femoroacetabular impingement. Bone Joint J 100–B(10):1275–1279 10.1302/0301-620X.100B10.BJJ-2018-0060.R1 30295531

[38] Patel RV et al (2020) Pelvic Tilt and range of motion in hips with femoroacetabular impingement syndrome. JAAOS-Journal Am Acad Orthop Surg 28(10):e427–e432 10.5435/JAAOS-D-19-00155 31599764

